# Biomarker-estimated flavan-3-ol intake is associated with lower blood pressure in cross-sectional analysis in EPIC Norfolk

**DOI:** 10.1038/s41598-020-74863-7

**Published:** 2020-10-21

**Authors:** Javier I. Ottaviani, Abigail Britten, Debora Lucarelli, Robert Luben, Angela A. Mulligan, Marleen A. Lentjes, Reedmond Fong, Nicola Gray, Philip B. Grace, Deborah H. Mawson, Amy Tym, Antonia Wierzbicki, Nita G. Forouhi, Kay-Tee Khaw, Hagen Schroeter, Gunter G. C. Kuhnle

**Affiliations:** 1 Mars, Inc., McLean, VA USA; 2 MRC Epidemiology Unit, University of Cambridge, Cambridge, UK; 3 Department of Public Health and Primary Care, University of Cambridge, Cambridge, UK; 4 School of Medical Sciences, Örebro University, Örebro, Sweden; 5 Department of Nutrition, UC Davis, Davis, CA USA; 6 Department of Food and Nutritional Sciences, University of Reading, Reading, UK; 7 LGC, Newmarket Road, Fordham, UK

**Keywords:** Risk factors, Hypertension, Epidemiology

## Abstract

Flavan-3-ols are a group of bioactive compounds that have been shown to improve vascular function in intervention studies. They are therefore of great interest for the development of dietary recommendation for the prevention of cardio-vascular diseases. However, there are currently no reliable data from observational studies, as the high variability in the flavan-3-ol content of food makes it difficult to estimate actual intake without nutritional biomarkers. In this study, we investigated cross-sectional associations between biomarker-estimated flavan-3-ol intake and blood pressure and other CVD risk markers, as well as longitudinal associations with CVD risk in 25,618 participants of the European Prospective Investigation into Cancer (EPIC) Norfolk cohort. High flavan-3-ol intake, achievable as part of an habitual diet, was associated with a significantly lower systolic blood pressure (− 1.9 (− 2.7; − 1.1) mmHg in men and − 2.5 (− 3.3; − 1.8) mmHg in women; lowest vs highest decile of biomarker), comparable to adherence to a Mediterranean Diet or moderate salt reduction. Subgroup analyses showed that hypertensive participants had stronger inverse association between flavan-3-ol biomarker and systolic blood pressure when compared to normotensive participants. Flavanol intake could therefore have a role in the maintenance of cardiovascular health on a population scale.

## Introduction

High blood pressure is a leading disease risk factor globally, and cardiovascular disease (CVD) is a main cause of death^1^. Therefore, the primary prevention of CVD remains of utmost importance, and changes in dietary factors have an important rôle. While dietary recommendations for primary disease prevention have focused mainly on dietary patterns and macronutrients^2^, increasing attention has been given to a group of non-nutritive dietary compounds, bioactives, that are thought to exert a physiological effect and to modulate disease risk^3^. Flavan-3-ols are a major class of dietary bioactives^4–6^, belonging to the group of polyphenolics, commonly found in tea, pome fruits, berries, cocoa-derived products and nuts. Accumulating evidence from dietary intervention studies shows that the intake of flavan-3-ols improves vascular function in healthy adults^6,7^. Indeed, multiple clinical dietary intervention studies have demonstrated flavanol-intake related cardiovascular health benefits by assessing physiological endpoints including blood pressure, flow-mediated arterial dilation, augmentation index, pulse wave velocity and arterial stiffness, as well as atherogenesis^8–10^. However, the data currently available are neither describing effects at sufficient scale nor were derived from long-term investigations and are thus insufficient provide the basis for population-based dietary guidance^6^. While ongoing large-scale clinical dietary intervention studies, such as COSMOS (NCT02422745^11^), with specific focus on cardiovascular disease risk and outcomes measures that include stroke, myocardial infarction and blood pressure, are intended to close this gap, large-scale observational studies can provide crucial information about associations between habitual flavan-3-ol intake and vascular health, especially as these studies usually include a wide range of different foods and beverages and are based on a more heterogeneous population representative of the general public.

Importantly, large-scale observational studies rely on the accurate assessment of intake. To date, all such studies aiming to estimate flavan-3-ol intake were based on self-reported dietary data, food frequency questionnaires (FFQs) or food diaries, in combination with food composition data. While this approach, in the absence of practicable alternatives, has been tacitly accepted as de facto standard^12–16^, it introduces significant limitations that substantially affect outcome and interpretation. Self-reported dietary data are subject to a number of limitations and have been demonstrated to be subject to systematic bias^17^. Detailed analyses, for example of protein and energy^18^, but also sugar intake^19^, have shown systematic under-reporting. While these methods can provide reliable data on dietary patterns and intake of individual foods, these limitations affect the ability to estimate intake of individual compounds. This is further exacerbated by the reliance on food composition data, which can only provide data on average food content and not the composition of the foods actually consumed. For flavan-3-ols but also other compounds found in foods, this approach introduces significant error due to the large variability of food composition^20^, the effects of processing^21^ and differences in bioavailability. Thus, the reliance on food composition data to estimate flavan-3-ol intake introduces a considerable measurement error: for example, the amount found in tea, one of the main dietary sources in the UK diet^22^, ranges from 10 to 330 mg/100 g^20^. This problem is made worse by the common reliance on FFQs, which do not provide sufficient detail on food intake to allow an accurate estimate of actual intake^23^.

In contrast, nutritional biomarkers, which are assessed by measuring the systemic presence of dietary compounds or their metabolites, have the potential to mitigate the above limitations and thus enable objective and accurate estimates of actual intake^24–26^. As biomarkers for estimating flavan-3-ol intake were not available previously, we developed and evaluated at scale nutritional biomarkers to estimate the intake of flavan-3-ols in general, based on the flavan-3-ol-derived microbial metabolite 5-3$^\prime$,4$^\prime$-dihydroxyphenyl-$\gamma$-valerolactone (gVLM)^27^, and one specific for (–)-epicatechin intake, based on structurally related (–)-epicatechin metabolites (SREM)^28^. The biomarkers derived from those metabolites, referred to as $\hbox {gVLM}_B$ and $\hbox {SREM}_B$, are surrogate biomarkers^29^ when assessed in spot urine and therefore allow people to be ranked according to their flavan-3-ol intake.

These novel biomarkers allow rigorous, and more objective and accurate investigations into associations between actual flavan-3-ol intake and health in observational cohorts at scales relevant to human populations. The primary objective of this study was to investigate cross-sectional associations between biomarker-estimated flavan-3-ol intake and blood pressure in more than 25,000 participants of the Norfolk cohort of the European Prospective Investigation into Cancer Study (EPIC-Norfolk). The secondary objectives of the study were to investigate cross-sectional associations with other cardio-vascular disease risk factors and prospective associations with cardio-vascular disease risk.

## Results

### Study population and biomarker

This study was based on data of 25,618 participants (14,026 women, 55%) of EPIC-Norfolk, after the exclusion of those lost to follow-up (2 women, 1 man), and those who withdrew consent (18). Table 1 shows a summary of the baseline characteristics of the study population; more detailed data, including information on missing data, are shown as Supplemental Information (Supplementary Tables 1 and 2). Biomarker concentrations were available for 24,152 participants (13,273 women, 55%). Using specific gravity, spot urine samples were adjusted for dilution (25), and these data were available for 21,812 participants (11,974 women, 55%), as specific gravity data were not available for all. Missing data were assumed to be missing at random and imputed using multiple imputations. The concentrations of both biomarkers, $\hbox {gVLM}_B$ for flavan-3-ols and $\hbox {SREM}_B$ for (–)-epicatechin, were correlated (Pearson’s $\rho$ = 0.45).

**Figure 1:**
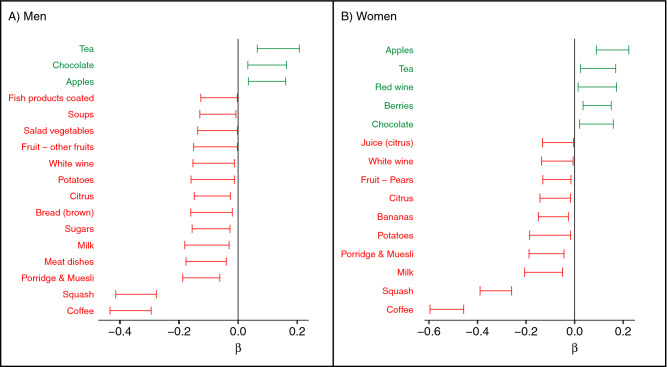
Association between biomarker-estimated flavan-3-ol intake ($\hbox {gVLM}_B$) and selected food groups from 7-day diaries. Biomarker-estimated intake was positively associated with tea, wine and apple intake, whereas there were inverse associations with coffee and squash (cordial) intake. $\beta$ are changes in specific gravity-adjusted biomarker concentration per SD change of respective reported food group weight, adjusted by total energy intake, social class, BMI and age; only food groups with statistically significant association (p < 0.05) are shown.

We have investigated the correlation between biomarker-estimated flavan-3-ol and self-reported food and flavan-3-ol intake. There were weak correlations (R$^2 < 0.2$) between flavan-3-ol biomarkers and the consumption of foods associated with flavan-3-ol intake in the diet of participants in EPIC-Norfolk (Fig. 1), and virtually no correlation with flavan-3-ol intake estimated using 7-day food diaries (R$^2$: 0.01 for $\hbox {gVLM}_B$ and 0.07 for $\hbox {SREM}_B$, adjusted for energy intake). These findings are consistent with the limitations of estimating bioactive-intake from self-reported dietary data described above, in particular the high variability in food composition.

**Table 1:** Baseline characteristics and disease incidence of 25,618 participants of EPIC Norfolk.

	All	Men	Women
n	25,618	11,592	14,026
Age (years)	58.7 (9.3)	59.1 (9.3)	58.4 (9.3)
BMI (kg/m$^2$)	26.4 (3.9)	26.5 (3.3)	26.2 (4.4)
Female (%)	14,026 (55%)	–	–
**Physical activity**			
Inactive	7853 (31%)	3579 (31%)	4274 (31%)
Moderately inactive	7344 (29%)	2853 (25%)	4491 (32%)
Moderately active	5773 (23%)	2657 (23%)	3116 (22%)
Active	4647 (18%)	2502 (22%)	2145 (15%)
**Smoking status**			
Current	2979 (12%)	1402 (12%)	1577 (11%)
Former	10,751 (42%)	6276 (55%)	4475 (32%)
Never	11,668 (46%)	3833 (33%)	7835 (56%)
Systolic BP (mmHg)	135 (18)	137 (18)	134 (19)
Diastolic BP (mmHg)	83 (11)	84 (11)	81 (11)
Cholesterol (mmol/L)	6.2 (1.2)	6.0 (1.1)	6.3 (1.2)
LDL (mmol/L)	4.0 (1.0)	3.9 (1.0)	4.0 (1.1)
HDL (mmol/L)	1.4 (0.4)	1.2 (0.3)	1.6 (0.4)
Triglycerides (mmol/L)	1.8 (1.1)	2.1 (1.2)	1.6 (1.0)
cRP (mg/L)	3.1 (6.3)	3.0 (6.0)	3.0 (5.9)
**Medication user**			
Lipid lowering drugs	377 (2%)	173 (2%)	204 (2%)
Anti-hypertensive drugs	4798 (19%)	2165 (19%)	2633 (19%)
**Biomarker**			
gVLM ($\upmu$mol/L)	9.5 (16.0)	10.5 (16.5)	8.7 (15.5)
gVLM ($\upmu$mol/L)$^{\text {a}}$	9.3 (15.7)	10.2 (16.2)	8.6 (15.3)
$\hbox {SREM}_B$ ($\upmu$mol/L)	2.0 (3.0)	2.2 (3.0)	1.8 (3.0)
$\hbox {SREM}_B$ ($\upmu$mol/L)$^{\text {a}}$	1.9 (2.9)	2.1 (2.9)	1.8 (2.9)
**Disease incidence**$^{\text {b}}$			
All CVD	13,969 (55%)	6907 (60%)	7062 (50%)
**Mortality**$^{\text {b}}$			
All cause	8030 (31%)	4277 (37%)	3753 (27%)
CVD	2613 (10%)	1474 (13%)	1139 (8%)

Data shown are mean (SD) or absolute number and proportion. More details, including number of missing data, are shown in Supplementary Tables 1 and 2.

$^{\text {a}}$Adjusted by specific gravity.

$^{\text {b}}$Number at end of follow-up.

### Cross-sectional associations between flavan-3-ol biomarker and blood pressure

**Figure 2:**
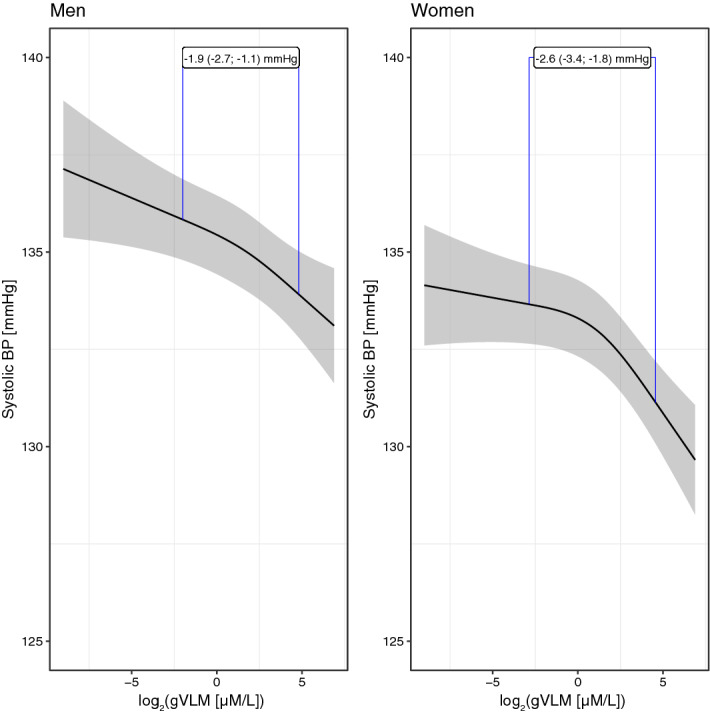
Association between biomarker estimated flavan-3-ol intake and systolic blood pressure. Predicted systolic blood pressure (95% confidence interval) in men (left, n=11,592) and women (right, n=14,026) adjusted for (model 5) age and BMI, smoking status, physical activity and social class, plasma vitamin C as marker of fruit and vegetable intake, tea and coffee intake, baseline health (self-reported diabetes mellitus, myocardial infarction, cerebrovascular accident), family history of myocardial infarction, use of anti-hypertensive or lipid-lowering drugs and menopausal status and hormone replacement therapy in women. The blue lines indicate the estimated differences in systolic blood pressure between low (10th percentile) and high (90th percentile) concentrations of the biomarker. Biomarker concentrations were adjusted by specific gravity.

The evidence available from a wide range of clinical intervention studies show that flavan-3-ols can have a vasculoprotective effect^6^, but this has never been shown on a large scale in a general population. We have therefore investigated the cross-sectional association between flavan-3-ol intake and blood pressure at baseline (Fig.  2). Table 2 shows consistent inverse associations between biomarkers of flavan-3-ol ($\hbox {gVLM}_B$) and (–)-epicatechin ($\hbox {SREM}_B$) intake and systolic blood pressure in all models tested. We have further investigated whether the biomarker merely acts as a surrogate marker of specific dietary patterns that are associated with blood pressure. Tea is a main dietary source of flavan-3-ol in EPIC-Norfolk^15^, and thus $\hbox {gVLM}_B$ and $\hbox {SREM}_B$ could both act as a marker of high tea or low coffee intake, as there was a strong inverse association between tea and coffee intake (Pearson’s $\rho$ = 0.4); similarly, fruits and vegetables can be an important contributor of flavan-3-ols. However, adjusting our data analysis additionally for tea and coffee intake, as well plasma vitamin C, as a surrogate marker of fruit and vegetable intake^30^, associations did not change materially (Table 2). When using $\hbox {gVLM}_B$ as biomarker of the intake of flavan-3-ol in general, the difference in systolic blood pressure between bottom and top decile of biomarker concentrations, the median of the bottom and top quintile, was − 1.9 (− 2.7; − 1.1) mmHg in men and − 2.5 (− 3.3; − 1.8) mmHg in women (model 5). Compared to the results using $\hbox {gVLM}_B$, the differences in blood pressure between the bottom and top decile were larger when using $\hbox {SREM}_B$ as specific biomarker of (–)-epicatechin intake, − 2.4 (− 3.3; − 1.5) mmHg in men and − 2.5 (− 3.6; − 2.0) mmHg in women. We found similar associations for diastolic blood pressure and biomarker-estimated flavan-3-ol intake, with a difference between the bottom and top decile of approximately 1 mmHg.

**Table 2:** Associations between biomarker-estimated flavan-3-ol intake and blood pressure.

	Men (n = 11,592)		Women$^{\text {b}}$ (n = 14,026)	
	Systolic BP (mm Hg)	Diastolic BP (mm Hg)	Systolic BP (mm Hg)	Diastolic BP (mm Hg)
**gVLM**_***B***_$^{\text {c}}$				
Model 0$^{\text {a}}$	− 1.6 (− 2.4; − 0.8)	− 1.0 (− 1.5; − 0.4)	− 1.8 (− 2.5; − 1.0)	− 1.1 (− 1.6; − 0.6)
Model 1	− 1.8 (− 2.6; − 1.0)	− 1.2 (− 1.7; − 0.7)	− 2.2 (− 3.0; − 1.5)	− 1.4 (− 1.9; − 0.9)
Model 2	− 1.8 (− 2.7; − 1.0)	− 1.2 (− 1.7; − 0.6)	− 2.3 (− 3.0; − 1.5)	− 1.4 (− 1.9; − 0.9)
Model 3	− 2.0 (− 2.8; − 1.2)	− 1.3 (− 1.8; − 0.7)	− 2.6 (− 3.3; − 1.8)	− 1.6 (− 2.0; − 1.1)
Model 4	− 1.8 (− 2.6; − 1.0)	− 1.2 (− 1.7; − 0.7)	− 2.3 (− 3.0; − 1.5)	− 1.4 (− 1.9; − 0.9)
Model 5	− 1.9 (− 2.7; − 1.1)	− 1.3 (− 1.8; − 0.7)	− 2.5 (− 3.3; − 1.8)	− 1.6 (− 2.0; − 1.1)
**SREM**_***B***_$^{\text {c}}$				
Model 0	− 1.8 (− 2.6; − 0.9)	− 0.7 (− 1.3; − 0.2)	− 1.3 (− 2.1; − 0.5)	− 0.3 (− 0.8; 0.2)
Model 1	− 2.0 (− 2.8; − 1.2)	− 0.9 (− 1.4; − 0.4)	− 1.9 (− 2.7; − 1.1)	− 0.8 (− 1.2; − 0.3)
Model 2	− 2.1 (− 2.9; − 1.2)	− 0.9 (− 1.4; − 0.4)	− 2.0 (− 2.7; − 1.2)	− 0.8 (− 1.3; − 0.3)
Model 3	− 2.5 (− 3.3; − 1.6)	− 1.2 (− 1.7; − 0.6)	− 2.9 (− 3.7; − 2.1)	− 1.2 (− 1.7; − 0.7)
Model 4	− 2.0 (− 2.8; − 1.2)	− 0.9 (− 1.4; − 0.4)	− 1.9 (− 2.7; − 1.1)	− 0.8 (− 1.3; − 0.3)
Model 5	− 2.4 (− 3.3; − 1.5)	− 1.2 (− 1.7; − 0.6)	− 2.8 (− 3.6; − 2.0)	− 1.2 (− 1.7; − 0.6)

Results shown are estimated differences (95% CI) between low (10th percentile) and high (90th percentile) biomarker concentrations, using multi-variable linear regression different statistical models.

$^{\text {a}}$Model 0: adjusted for age; model 1: additionally adjusted for BMI; model 2: additionally adjusted for smoking status, physical activity and social class; model 3: additionally adjusted plasma vitamin C as marker of fruit and vegetable intake, tea and coffee intake; model 4: model 2, additionally adjusted for baseline health (self-reported diabetes mellitus, myocardial infarction, cerebrovascular accident), family history of myocardial infarction, use of anti-hypertensive or lipid-lowering drugs; model 5: model 3, additionally adjusted for baseline health (self-reported diabetes mellitus, myocardial infarction, cerebrovascular accident), family history of myocardial infarction, use of anti-hypertensive or lipid-lowering drugs.

$^{\text {b}}$Additionally adjusted for menopausal status and hormone replacement therapy.

$^{\text {c}}$Biomarker concentrations were adjusted by specific gravity.

### Association between flavanol intake and other cardiovascular disease risk markers

We investigated cross-sectional associations between flavan-3-ol and (–)-epicatechin biomarker and other established CVD risk markers (blood lipids and c-reactive protein). There were small differences in blood lipids between the bottom and top decile of flavan-3-ol intake assessed with both $\hbox {SREM}_B$ and $\hbox {gVLM}_B$, with participants in the top decile of biomarker having lower blood cholesterol and LDL concentrations, but higher triglycerides. The associations were very similar for $\hbox {SREM}_B$ and $\hbox {gVLM}_B$. We did not find any associations of the flavanol biomarkers with c-reactive protein concentration (Table 3).

**Table 3:** Associations between biomarker-estimated flavan-3-ol intake and CVD risk markers.

	Cholesterol (mmol/L)	HDL (mmol/L)	LDL (mmol/L)	Triglycerides (mmol/L)	CRP (mg/L)
	**Men (n = 11,592)**				
**gVLM**_***B***_$^{\text {c}}$					
Model 0$^{\text {a}}$	− 0.1 (− 0.1; 0.0)	0.0 (0.0; 0.0)	− 0.1 (− 0.1; 0.0)	0.1 (0.0; 0.1)	− 0.1 (− 0.5; 0.2)
Model 1	− 0.1 (− 0.1; 0.0)	0.0 (0.0; 0.0)	− 0.1 (− 0.1; 0.0)	0.1 (0.0; 0.1)	− 0.2 (− 0.5; 0.2)
Model 2	− 0.1 (− 0.1; 0.0)	0.0 (0.0; 0.0)	− 0.1 (− 0.1; 0.0)	0.1 (0.0; 0.1)	− 0.2 (− 0.5; 0.1)
Model 3	− 0.1 (− 0.1; 0.0)	0.0 (0.0; 0.0)	− 0.1 (− 0.1; 0.0)	0.0 (0.0; 0.1)	− 0.2 (− 0.6; 0.1)
Model 4	− 0.1 (− 0.1; 0.0)	0.0 (0.0; 0.0)	− 0.1 (− 0.1; 0.0)	0.1 (0.0; 0.1)	− 0.2 (− 0.5; 0.2)
Model 5	− 0.1 (− 0.1; 0.0)	0.0 (0.0; 0.0)	− 0.1 (− 0.1; 0.0)	0.1 (0.0; 0.1)	− 0.2 (− 0.5; 0.1)
**SREM**_***B***_$^{\text {c}}$					
Model 0	− 0.1 (− 0.2; − 0.1)	0.0 (0.0; 0.0)	− 0.2 (− 0.2; − 0.1)	0.1 (0.1; 0.2)	0.2 (− 0.2; 0.5)
Model 1	− 0.1 (− 0.2; − 0.1)	0.0 (0.0; 0.0)	− 0.2 (− 0.2; − 0.1)	0.1 (0.1; 0.2)	0.1 (− 0.2; 0.5)
Model 2	− 0.1 (− 0.2; − 0.1)	0.0 (0.0; 0.0)	− 0.2 (− 0.2; − 0.1)	0.1 (0.1; 0.2)	0.1 (− 0.3; 0.4)
Model 3	− 0.1 (− 0.1; 0.0)	0.0 (0.0; 0.0)	− 0.1 (− 0.2; − 0.1)	0.1 (0.0; 0.2)	0.0 (− 0.4; 0.3)
Model 4	− 0.1 (− 0.2; − 0.1)	0.0 (0.0; 0.0)	− 0.2 (− 0.2; − 0.1)	0.1 (0.1; 0.2)	0.1 (− 0.3; 0.4)
Model 5	− 0.1 (− 0.1; 0.0)	0.0 (0.0; 0.0)	− 0.1 (− 0.2; − 0.1)	0.1 (0.1; 0.2)	0.0 (− 0.3; 0.4)
	**Women**$^{\text {b}}$ **(n = 14,026)**				
**gVLM**_***B***_$^{\text {c}}$					
Model 0	− 0.1 (− 0.1; 0.0)	0.0 (− 0.1; 0.0)	− 0.1 (− 0.1; 0.0)	0.1 (0.1; 0.2)	0.3 (0.0; 0.6)
Model 1	− 0.1 (− 0.1; 0.0)	0.0 (0.0; 0.0)	− 0.1 (− 0.1; 0.0)	0.1 (0.0; 0.1)	0.2 (− 0.1; 0.5)
Model 2	− 0.1 (− 0.1; 0.0)	0.0 (0.0; 0.0)	− 0.1 (− 0.1; 0.0)	0.1 (0.0; 0.1)	0.2 (− 0.1; 0.5)
Model 3	− 0.1 (− 0.1; 0.0)	0.0 (0.0; 0.0)	− 0.1 (− 0.1; 0.0)	0.1 (0.0; 0.1)	0.1 (− 0.2; 0.4)
Model 4	− 0.1 (− 0.1; 0.0)	0.0 (0.0; 0.0)	− 0.1 (− 0.1; 0.0)	0.1 (0.0; 0.1)	0.2 (− 0.1; 0.5)
Model 5	− 0.1 (− 0.1; 0.0)	0.0 (0.0; 0.0)	− 0.1 (− 0.1; 0.0)	0.1 (0.0; 0.1)	0.1 (− 0.2; 0.4)
**SREM**_***B***_$^{\text {c}}$					
Model 0	0.0 (− 0.1; 0.1)	0.0 (0.0; 0.0)	0.0 (− 0.1; 0.0)	0.2 (0.1; 0.2)	0.4 (0.1; 0.7)
Model 1	0.0 (− 0.1; 0.0)	0.0 (0.0; 0.0)	− 0.1 (− 0.1; 0.0)	0.1 (0.1; 0.2)	0.3 (0.0; 0.6)
Model 2	0.0 (− 0.1; 0.0)	0.0 (0.0; 0.0)	− 0.1 (− 0.1; 0.0)	0.1 (0.1; 0.2)	0.3 (0.0; 0.6)
Model 3	0.0 (− 0.1; 0.1)	0.0 (0.0; 0.0)	− 0.1 (− 0.1; 0.0)	0.1 (0.1; 0.1)	0.1 (− 0.3; 0.4)
Model 4	0.0 (− 0.1; 0.0)	0.0 (0.0; 0.0)	− 0.1 (− 0.1; 0.0)	0.1 (0.1; 0.2)	0.3 (0.0; 0.6)
Model 5	0.0 (− 0.1; 0.1)	0.0 (0.0; 0.0)	− 0.1 (− 0.1; 0.0)	0.1 (0.1; 0.1)	0.1 (− 0.2; 0.4)

Results shown are estimated differences (95% CI) between low (10th percentile) and high (90th percentile) biomarker concentrations, using multi-variable linear regression different statistical models.

$^{\text {a}}$Model 0: adjusted for age; model 1: additionally adjusted for BMI; model 2: additionally adjusted for smoking status, physical activity and social class; model 3: additionally adjusted plasma vitamin C as marker of fruit and vegetable intake, tea and coffee intake; model 4: model 2, additionally adjusted for baseline health (self-reported diabetes mellitus, myocardial infarction, cerebrovascular accident), family history of myocardial infarction, use of anti-hypertensive or lipid-lowering drugs; model 5: model 3, additionally adjusted for baseline health (self-reported diabetes mellitus, myocardial infarction, cerebrovascular accident), family history of myocardial infarction, use of anti-hypertensive or lipid-lowering drugs.

$^{\text {b}}$Additionally adjusted for menopausal status and hormone replacement therapy.

$^{\text {c}}$Biomarker concentrations were adjusted by specific gravity.

### Association with CVD incidence and mortality

High flavan-3-ol intake was associated with lower blood pressure and an overall better blood lipid profile and may therefore tenably affect overall CVD risk and mortality. Thus, in a secondary analysis, we have investigated associations between $\hbox {gVLM}_B$ as biomarker of flavan-3-ol intake and CVD risk and CVD and all-cause mortality. After a median of 19.5 (IQR 17.9–20.9) years of follow-up, 8030 (31%) participants had died and 13,969 (55%) had developed a cardiovascular disease. Overall, there were no consistent, statistically significant associations between flavan-3-ol biomarker and CV disease incidence or all cause or CVD mortality (Table 3).

#### Subgroup and sensitivity analyses

**Figure 3:**
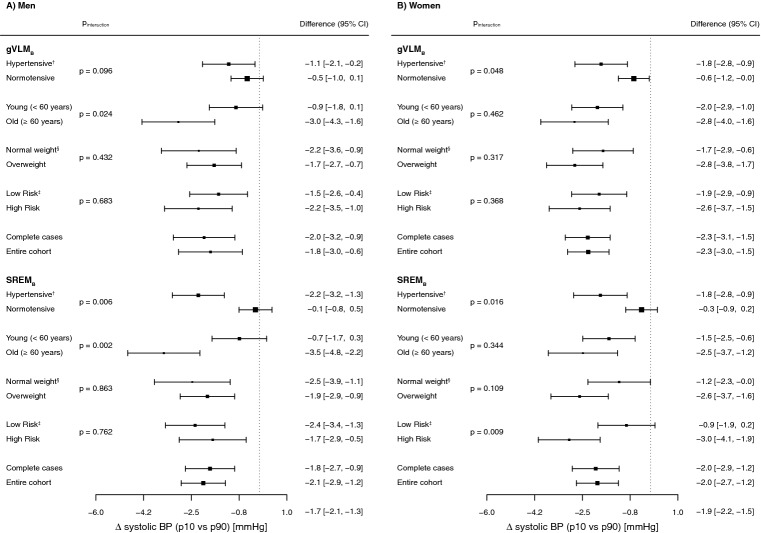
Subgroup- and sensitivity analysis, comparing estimated differences in systolic blood pressure between sex-specific bottom (p10) and top (p90) decile of biomarker-estimated flavan-3-ol intake. Models were adjusted by age, BMI, smoking status, physical activity and social class, and additionally for menopausal status and hormone-replacement therapy for women. $^\dag$Hypertensive: systolic BP $\ge$ 140 mmHg; $^\S$normal weight: BMI $\le$ 25 kg/m$^2$; $^\ddag$low risk: no baseline prevalence of diabetes or heart disease, no family history of heart disease, no anti-hypertensive or lipid-lowering drug use at baseline.

We conducted sub-group and sensitivity analyses to further investigate the associations between biomarker-estimated flavan-3-ol intake and blood pressure (Fig. 3). We aimed at investigating whether or not pre-existing CVD or CVD risk would affect the association between systolic blood pressure and biomarker-estimated flavan-3-ol intake. Therefore, we compared the difference in systolic blood pressure between the top and bottom decile of the flavan-3-ol biomarker in participants with and without pre-existing CVD or CVD risk (age, hypertension, overweight, and prevalent CVD at baseline). Hypertensive participants had stronger inverse association between flavan-3-ol biomarker and systolic blood pressure when compared to normotensive participants, in particular when using $\hbox {SREM}_B$ as biomarker. Similar differences were observed between hypertensive and normotensive women using $\hbox {gVLM}_B$-estimated flavan-3-ol intakes. In addition, the difference in systolic blood pressure between the top and bottom decile of the $\hbox {SREM}_B$- and $\hbox {gVLM}_B$-estimated flavan-3-ol intake in older men (>60 years) was greater than that in younger men. Finally, significant differences were also observed in women with low and high risk of CVD using $\hbox {SREM}_B$ as biomarker of flavan-3-ol intake.

**Table 4:** Associations between $\hbox {gVLM}_B$ as biomarker of flavan-3-ol intake and CVD incidence and CVD and all-cause mortality.

	Men (n = 11,592)		Women$^{\text {b}}$ (n = 14,026)	
	All-cause	CVD	All-cause	CVD
**Incidence**				
Model 0$^{\text {a}}$	–	1.04 (0.97; 1.11)	–	1.06 (0.99; 1.13)
Model 1	–	1.03 (0.96; 1.10)	–	1.03 (0.97; 1.10)
Model 2	–	1.02 (0.95; 1.09)	–	1.02 (0.95; 1.09)
Model 3	–	1.01 (0.95; 1.08)	–	1.01 (0.94; 1.07)
Model 4	–	1.04 (0.98; 1.11)	–	1.02 (0.96; 1.09)
Model 5	–	1.04 (0.98; 1.11)	–	1.01 (0.95; 1.08)
**Mortality**$^{\text {c}}$				
Model 0	0.99 (0.91; 1.08)	0.99 (0.86; 1.15)	1.00 (0.92; 1.09)	1.02 (0.87; 1.20)
Model 1	0.98 (0.90; 1.07)	0.99 (0.85; 1.14)	1.00 (0.91; 1.09)	1.01 (0.86; 1.19)
Model 2	0.97 (0.89; 1.05)	0.98 (0.84; 1.13)	0.97 (0.89; 1.06)	0.99 (0.85; 1.16)
Model 3	0.96 (0.88; 1.05)	0.97 (0.83; 1.12)	0.97 (0.88; 1.05)	1.00 (0.86; 1.18)
Model 4	0.98 (0.90; 1.07)	1.01 (0.88; 1.17)	0.97 (0.89; 1.06)	1.01 (0.86; 1.18)
Model 5	0.98 (0.90; 1.07)	1.01 (0.87; 1.17)	0.97 (0.89; 1.06)	1.03 (0.87; 1.20)

Results shown are estimated differences (HR, 95% CI) between low (10th percentile) and high (90th percentile) biomarker concentrations, using different statistical models.

$^{\text {a}}$Model 0: stratified by age-decade; model 1: additionally adjusted for BMI; model 2: additionally adjusted for smoking status, physical activity and social class; model 3: additionally adjusted plasma vitamin C as marker of fruit and vegetable intake, tea and coffee intake; model 4: model 2, additionally adjusted for baseline health (self-reported diabetes mellitus, myocardial infarction, cerebrovascular accident), family history of myocardial infarction, use of anti-hypertensive or lipid-lowering drugs; model 5: model 3, additionally adjusted for baseline health (self-reported diabetes mellitus, myocardial infarction, cerebrovascular accident), family history of myocardial infarction, use of anti-hypertensive or lipid-lowering drugs.

$^{\text {b}}$Additionally adjusted for menopausal status and hormone replacement therapy.

$^{\text {c}}$Biomarker concentrations were adjusted by specific gravity.

## Discussion

In participants of EPIC-Norfolk, a large cohort representative of the older general public in England^31^, high flavan-3-ol intake was associated with a significantly lower systolic and diastolic blood pressure and was inversely associated with blood lipids. We did not observe consistent, statistically significant associations between biomarkers of flavan-3-ol intake and CVD incidence or mortality.

This study was enabled by recently developed nutritional biomarkers, $\hbox {gVLM}_B$ and $\hbox {SREM}_B$, that allow for estimating specifically the intake of flavan-3-ols. We have shown previously that $\hbox {gVLM}_B$ and $\hbox {SREM}_B$ reflect actual intake of flavan-3-ols and (–)-epicatechin respectively, yet importantly here we found only a weak correlation between biomarker and self-reported food intake^27,28^. This finding strongly supports our previous findings of the impact of the high variability in food composition on self-reported dietary assessment methods^23^. The high variability in food composition of many of the main sources of dietary flavan-3-ol make an accurate estimate without an analysis of the actual food consumed virtually impossible. For example, food composition data^20^ for black tea give a range of flavan-3-ol content of 3–64 mg/100 mL, and thus five cups of tea can contain between 23 and 480 mg of flavan-3-ols. Thus, a person consuming a single cup of tea with high flavan-3-ol content consumes considerably more flavan-3-ols than a person consuming five cups of tea with low flavan-3-ol content. A wide variability in food composition has been reported for other foods, too. Indeed, even for foods grown on the self-same plant, up to 2.5-fold differences for some nutrients have been observed^32^. In contrast, the objective nutritional biomarkers used in this study take into account the diversity in the foods consumed and differences in bioavailability as they rely on the systemic presence of the respective compounds. The inclusion of the main dietary sources of flavan-3-ols and (–)-epicatechin in our statistical models (i.e. tea as well as fruit and vegetables^22^) did not affect outcomes (Table 2, models 3 and 5), which strongly supports the notion that the data provided by the nutritional biomarkers is related to the actual intake of bioactive compound and does not just reflect dietary patterns.

The two different biomarkers of flavan-3-ol intake used here ($\hbox {gVLM}_B$ and $\hbox {SREM}_B$), enabled estimating the dietary intake of flavan-3-ols^27^ and the flavan-3-ol (–)-epicatechin^28^, respectively. The differences between the effect sizes estimated with either biomarker were negligible (Tables 2 and 3), and this can to some extent be explained by the correlation between the two biomarkers. However, the two biomarkers used in this study originate from two distinct metabolic pathways, $\hbox {gVLM}_B$ from the catabolism of flavan-3-ols in the gut microbiome, $\hbox {SREM}_B$ from phase II biotransformation reactions of (–)-epicatechin. Thus, these results pose the question as to whether the observed association between flavan-3-ol intake and blood pressure could be explained by the specific intake of (–)-epicatechin, as it is the only compound measured with both biomarkers. In this context, previous intervention studies have established a vascular effect for (–)-epicatechin^33^, and showed that only (–)-epicatechin, but no other type of flavan-3-ols exert such activity^34^. While larger controlled dietary intervention studies are necessary to establish the actual bioactive compound and mode of action, our results clearly show an inverse association between flavan-3-ols, including (–)-epicatechin, and blood pressure, and thereby contribute to the data available to investigate a causal effect. While the specific molecular mechanisms that underlie the cardiovascular effects of flavanols are still under investigation, currently published work in this context indicates that flavanols mediate a range of effects on the cellular-/molecular level that impact on endothelial function, nitric oxide-dependent arterial dilation, thrombogenic responses, and processes related to vascular inflammation, angiogenesis, and endothelial repair^8–10,35–37^.

In comparison with the observed associations with blood pressure, the associations with blood lipids were rather modest. However, they were of a similar magnitude and direction than those observed in the Minnesota Green Tea Trial (MGTT^38^), even though flavan-3-ol intakes in EPIC-Norfolk were considerably lower.

### Impact on health

In this study, we could demonstrate significant inverse associations between biomarkers of flavan-3-ol and (–)-epicatechin intake and blood pressure at baseline. However, there were no consistent, statistically significant associations with CVD risk or mortality (CVD related and all-cause) (Table 4). This can be explained by the magnitude of difference in systolic blood pressure observed, which would not be expected to have significant impact on individual CVD risk (approximately 0.2 percentage points reduction in 10-year CVD risk based on QRISK 3^39^).

The difference in systolic blood pressure observed here between low and high biomarker concentration in the cross-sectional analysis (approximately 2 mmHg) is similar to the reduction in blood pressure observed in dietary intervention studies^7^. This difference is comparable to those observed with a Mediterranean diet in the PREDIMED trial (1.5 mmHg^40^) or a moderate reduction in salt intake in the DASH-Sodium trial (2.1 mmHg, high to intermediate sodium intake^41^), and could have considerable impact on health at a population scale. However, large-scale dietary intervention studies such as COSMOS (NCT02422745^11^) are required to confirm whether the observed differences in blood pressure can be explained by differences in flavan-3-ol intake.

**Figure 4:**
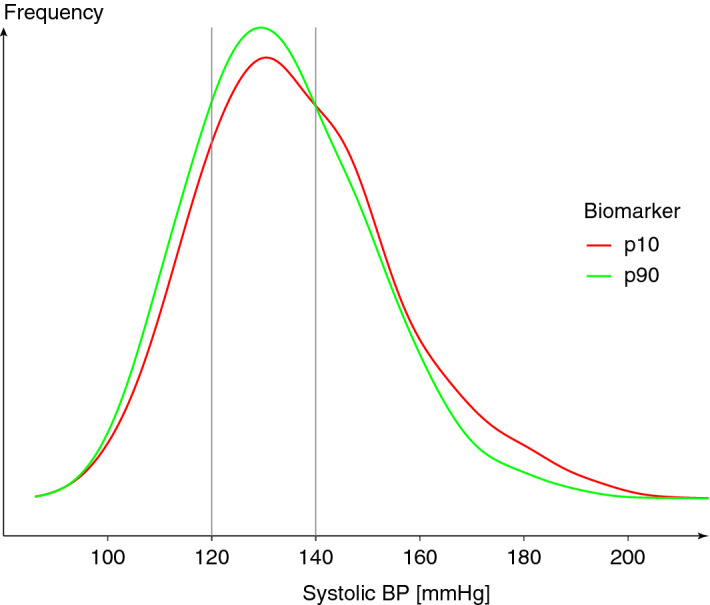
Distribution of systolic blood pressure of participants of EPIC Norfolk in the bottom (p10) and top (p90) decile of flavan-3-ol intake (estimated by $\hbox {gVLM}_B$). Approximately 40% of participants in the bottom decile (p10) were hypertensive or pre-hypertensive (systolic blood pressure *geq* 140 mmHg), compared to 33% in the top decile.

A subgroup analyses showed that the association between intake and blood pressure was strongest among participants at higher risk of developing cardiovascular diseases, in particular older participants and those with existing hypertension, confirming results from previous small-scale dietary intervention studies^7^. The association between intake and blood pressure therefore follows a progressive model^42^, where the strongest effect size is found in those with higher blood pressure (Fig. 4). Such a model has also been observed in other dietary interventions such as the effect of potassium^43^ or sodium^44^ intake on blood pressure or the DASH diet^45^. In such a model, even a small reduction in blood pressure can have a considerable impact on morbidity and mortality on a population scale as it reduces the prevalence of hypertension and pre-hypertension, and thus the number of people at higher risk of CVD. Indeed, a reduction of 3 mmHg systolic blood pressure can be translated into a reduction in all-cause mortality by 3%^42,46^. Flavanol intake could therefore have a role in the maintenance of cardiovascular health on a population scale.

**Figure 5:**
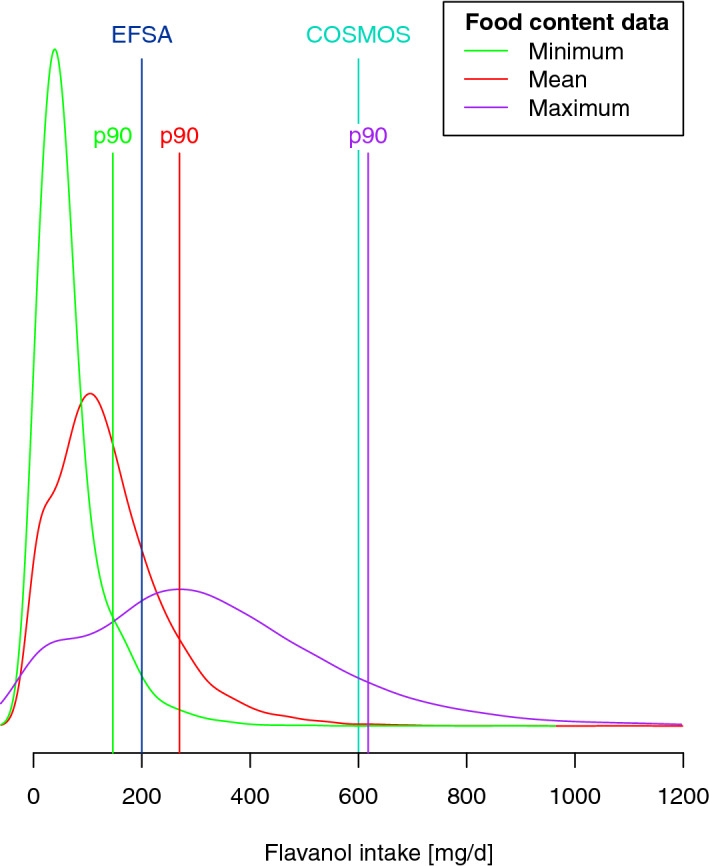
Distribution of flavan-3-ol and (–)-epicatechin intake in EPIC Norfolk, estimated using 7-day food diaries and minimum (green), mean (red) and maximum (purple) food composition data^20^. The graph indicates the 90th percentile (p90) used as high intake in this study (146 mg/day when using minimum food content data, 270 mg/day when using mean food consumption data as is common practice and 618 mg/day when using maximum food composition data), as well as the amounts used for the EFSA approved health claim (200 mg/day)^47^ and the COSMOS study (600 mg/day)^11^.

### Nutritional interpretation

The differences in blood pressure observed in this study were seen in a population with relatively high habitual intakes of flavan-3-ol and not any supplementation as has been used in intervention studies. In this context, it is noteworthy that, while representative of the UK population, the diet of participants of EPIC-Norfolk regarding flavan-3-ol intake may not be representative of countries without a strong tea culture, including countries in continental Europe and the US^15^. Compared to EPIC-Norfolk, it is therefore possible that a larger fraction of the population in countries without a tea culture could benefit from increasing the intake of flavan-3-ol in their diets. In addition, the question is still open as to whether or not the intake of flavan-3-ols beyond the ranges in the EPIC-Norfolk population would be associated with a further reduction in blood pressure. In any case, the large variability of flavan-3-ol content in foods precludes the identification of a diet that would result in a given increase of flavan-3-ol intake. While incorporating foods and beverages, such as tea, apples and berries, would probably increase intake of flavan-3-ols in the diet, it will depend not only on the type of food, but also on the actual product variety (species) consumed, the manufacturing conditions in which that product was generated and preserved and mode of preparation. This makes more specific recommendations impossible.

The biomarkers used in this study were surrogate biomarkers^29^. They are used to rank participants according to intake, but it is not possible to use them to calculate the actual amount of flavan-3-ol consumed. In order to estimate the amount consumed by participants in the top decile of intake, we have therefore used data from 7-day food diaries and calculated flavan-3-ol intakes using not only mean food content, as is common practice, but the entire range of reported food content (Fig. 5). Participants in the top decile of flavan-3-ol intake consumed at least 146 mg/day when using minimum, and 618 mg/day when using maximum food content (260 mg/day when using mean food content data) of flavan-3-ols, and at least 4 mg/day (minimum food content) or 138 mg/day (maximum food content) of (–)-epicatechin (36 mg/day when using mean food content data). While these figures provide an initial estimation, further efforts are needed to obtain a more precise number if these data are expected to be used for the development of dietary recommendations of flavan-3-ols as bioactive.

### Strengths and limitations

The EPIC-Norfolk cohort is ideally placed to investigate potential associations between flavan-3-ol intake and cardiovascular disease risk factors, not only because of its size, but also because it is set in a population with habitually high flavan-3-ol intake^22^. The main strength of the study is the use of robustly evaluated nutritional biomarkers to estimate flavan-3-ol^27^ and (–)-epicatechin^28^ intake and the use of 7-day-food diaries for dietary data. A limitation of the study is the reliance on a single spot-urine sample, as multiple samples would have provided a more representative estimation of habitual intake. The plasma half-life of gVLM, approximately 6 h, is sufficient to achieve steady-state like conditions with regular consumption^27^. As the main source of flavan-3-ol intake in a UK cohort is tea^22^, which is consumed regularly in a UK population, it can provide information on longer term intake. The half-life of SREM is considerably shorter, 2 h, and the biomarker therefore reflects mainly short-term intake. Further limitations are the largely cross-sectional nature and the inability to derive actual intake from surrogate biomarkers, only allowing the ranking of participants according to intake^29^.

### Conclusions

This study demonstrates the importance of nutritional biomarkers to estimate intake of plant bioactives to investigate associations between intake and disease risk, as only biomarkers can provide reliable information on actual bioactive intake. It also raises the important question of the impact of the variability in food composition on nutritional research and dietary assessment. The results of our study show a significant and biomedically relevant inverse association between biomarkers of flavan-3-ol intake and blood pressure in a free-living general population with a wide range of flavan-3-ol intake from their habitual diet. The observed difference is approximately comparable to that of adherence with the Mediterranean Diet or moderate salt reduction, and likely to have a considerable impact on a population scale. In the context of an ageing population and increased prevalence of chronic diseases, these findings hold promise for the prevention of cardiovascular disease through dietary approaches.

## Methods

### Study population

Between 1993 and 1997, 30,447 women and men aged between 40 and 75 years were recruited for the Norfolk cohort of the European Prospective Investigation into Cancer and Nutrition (EPIC) study, and 25,633 attended a health examination^31^. Health and lifestyle characteristics, including data on smoking, social class and family medical history, were assessed by questionnaire. Height and weight measurements were collected following a standardized protocol by trained research nurses. Physical activity, representing occupational and leisure activity, was assessed using a validated questionnaire^48^. Blood pressure was measured by using a non-invasive oscillometric blood pressure monitor (Acutorr; Datascope Medical, Huntingdon, UK; validated against sphygmomanometers every 6 months) after the participant had been seated in a comfortable environment for 5 min. The arm was horizontal and supported at the level of the mid-sternum; the mean of two readings was used for analysis. Non-fasting blood samples were taken by venepuncture and stored in serum tubes in liquid nitrogen. Serum levels of total cholesterol were measured on fresh samples with the RA 1000 autoanalyzer (Bayer Diagnostics, Basingstoke, UK). Plasma vitamin C was measured using a fluorometric assay as described previously^49^. Non-fasting spot urine samples were collected during the health examination and stored at − 20 $^\circ$C until analysis. Samples were collected throughout the day, and there were no consistent associations between collection time and biomarker concentration. Diet was assessed by 7-day diary (7DD), whereby the first day of the diary was completed as a 24-h recall (24HDR) with a trained interviewer and the remainder completed during subsequent days. Diary data were entered, checked and calculated using the in-house dietary assessment software DINER (Data into Nutrients for Epidemiological Research) and DINERMO^50^. Flavan-3-ol intake (the sum of epicatechin, catechin, epicatechin-3-*O*-gallate, catechin-3-*O*-gallate and proanthocyanidins) was estimated as described previously^15^; minimum and maximum estimated flavan-3-ol intake was estimated using the minimum and maximum food content data provided by Phenol Explorer und USDA databases^20^.

The study was approved by the Norwich Local Research Ethics Committee and all participants gave written, informed consent and all methods were carried out in accordance with relevant guidelines and regulations.

### Flavan-3-ol biomarker

We have used two different biomarkers to estimate flavan-3-ol intake, one based on the flavan-3-ol-derived microbial metabolite 5-3$^\prime$,4$^\prime$-dihydroxyphenyl-$\gamma$-valerolactone (gVLM)^27^, and one based on structurally related (–)-epicatechin metabolites (SREM)^28^: $\hbox {gVLM}_B$ that includes the metabolites 5-(3$^\prime$,4$^\prime$-dihydroxyphenyl)-$\gamma$-valerolactone-3$^\prime$-*O*-glucuronide (gVL3G) and 5-(3$^\prime$,4$^\prime$-dihydroxyphenyl)-$\gamma$-valerolactone-3$^\prime$-sulphate (gVL3S), and $\hbox {SREM}_B$ that includes the metabolites (–)-epicatechin-3$^\prime$-glucuronide (E3G), (–)-epicatechin-3$^\prime$-sulfate (E3S) and 3$^\prime$-*O*-methyl-(–)-epicatechin-5-sulfate (3Me5S). $\hbox {gVLM}_B$ are specific for estimating the intake of flavan-3-ols in general, including (±)-epicatechin, (±)-catechin, (±)-epicatechin-3-*O*-gallate, (±)-catechin-3-*O*-gallate and procyanidins and excluding the flavan-3-ols gallocatechin, epigallocatechin, gallocatechin-3-*O*-gallate, epigallocatechin-3-*O*-gallate, theaflavins and thearubigins^27^. $\hbox {SREM}_B$ are specific for (–)-epicatechin intake^28^. Spot urine samples were collected during the baseline health examination and stored in glass bottles at 20 $^\circ$C until analysis. Stability analyses confirmed that biomarkers are stable under these conditions^28^. Samples were analysed in random order using the method described previously^27,28^, with automated sample preparation (Hamilton Star robot; Hamilton, Bonaduz, Switzerland). Briefly, 60 $\upmu$L spot urine sample and internal standard solutions (2.5 $\upmu$M $^{13}\hbox {C}_2\hbox {D}_2$-5-(3$^\prime$,4$^\prime$-dihydroxyphenyl)-$\gamma$-valerolactone-3$^\prime$-sulphate, $\hbox {D}_2$/$\hbox {D}_3$-epicatechin-3$^\prime$-$\beta$-D-glucuronide, 50:50 mix)) were combined, filtered (Impact Protein Precipitation filter plate, Phenomenex, Macclesfield, UK; centrifuged for 2 min at 500 $\times$ *g* at room temperature and stored at 20 $^\circ$C until analysis. Samples were then separated by liquid chromatography (Acquity, Waters, Elstree, UK) using a C18 column (Kinetex C18 200 $\times$ 2.1 mm, 1.7 $\upmu$m, with 0.5 $\upmu$m Krudcatcher, Phenomenex, Macclesfield, UK) and detected by electrospray ionisation tandem mass spectrometry (Applied Biosystems API 4000, Sciex, Warrington, UK) in negative ion mode. The spray voltage was − 4500 V and the source temperature was 600 $^\circ$C. Samples were quantified using calibration standards prepared in flavan-3-ol-metabolite free urine samples using the peak area ratio of analyte and internal standard. Each batch included two replicates of quality control samples with three different concentrations: low QC (0.3 $\upmu$M), medium QC (2.5 $\upmu$M for $\hbox {SREM}_B$, 25 $\upmu$M for $\hbox {gVLM}_B$) and high QC (3.8 $\upmu$M for $\hbox {SREM}_B$, 38 $\upmu$M for $\hbox {gVLM}_B$) and usual acceptance criteria for each batch were that at least one QC at each concentration and four out of the six QCs were within 15% of the theoretical concentration. The accuracy of the method was better than 5%, precision of the method (%CV) below 12% for all analytes. The results for 224 randomly inserted duplicate samples showed a high correlation (R$^2$ = 0.92) with a mean difference of 1 (95% CI − 0.04; 6.9) $\upmu$mol/L. We did not observe any time-dependent change in method performance.

Concentrations below the lower limit of quantification (LLOQ, 0.1 $\upmu$M) were used for the analysis to avoid the bias of substituting a range of values by a single value. Concentrations below the limit of detection were assigned a value of 0.001 $\upmu$M. Concentrations were adjusted by specific gravity for dilution as the endpoint of the analysis, systolic blood pressure, was strongly correlated with urinary creatinine^51^.

### Incident CVD events and mortality

All participants were followed up for fatal and nonfatal CVD events, and the present study includes events until 31 March 2016, covering a median follow-up time of 19.5 (IQR 17.9; 20.9) years. Cause-specific hospital admission was determined via ENCORE (East Norfolk Commission Record, the hospital admissions database kept by the East Norfolk Health Commission)^52^ with the individuals’ unique National Health Service (NHS) number. All individuals were flagged by the UK Office of National Statistics (ONS) for death certification and trained nosologists coded death certificates according to the International Classification of Disease (ICD). The disease endpoints of this study was the first CVD event [defined as ICD 410–448 (ICD 9) or ICD I10–I79 (ICD 10)].

### Data analysis

Data analyses were carried out using R 3.6^53^, using the packages *rms*^54^ for regression analyses, *ggplot2*^55^ and *gridExtra*^56^ for the generation of graphics. Regression analyses were conducted using the *fit.mult.impute* function with either *ols* (cross-sectional analyses) or *cph* (prospective analyses) as regression function. We have used the Wald statistics calculated by the *rms* *anova* function to investigate the relationship between dependent and independent variables, and to test for linearity. tableone^57^ was used to prepare tables. Unless indicated otherwise, results are shown with 95% confidence intervals.

### Descriptive statistics

Descriptive characteristics of the study population were summarised using mean (standard deviation) for continuous variables and frequency (percentage) for categorical variables.

### Missing values

Missing values (Supplemental Table 1) were assumed to be missing at random and were imputed using multiple imputation using the *aregImpute* algorithm^54^, which uses predictive mean matching with optional weighted probability sampling. We have used different imputed datasets: for the analysis of cross-sectional associations with the primary endpoint (blood pressure), for the analysis with cross-sectional associations with the secondary endpoints (blood lipids), and one for each prospective association with disease endpoints and mortality. In each imputation, we created 50 imputed, using restricted cubic splines (3 knots) and not assuming linearity, including all variables used in the final analysis. For prospective analyses, we have also included the Nelson-Aalen estimator^58^. There were no meaningful differences when comparing results obtained from the full data set with complete cases analyses in cross-sectional data (Supplementary Tables 3 and 4).

### Data transformation

Biomarker data were positively skewed (log-normal distribution) and therefore log2-transformed data were used for all analyses. Restricted cubic splines (3 knots, outer quantiles 0.1 and 0.9; using the *rcs* function^54^) were used for all continuous variables unless indicated otherwise.

### Association between biomarker and self-reported intake

Associations between biomarker-estimated flavan-3-ol intake and different food groups were investigated using multivariable regression analysis, using z-scores of self-reported food group intake (n = 96) as independent variables. Models were adjusted for age, BMI, social class and energy intake, and stratified by sex.

### Cross-sectional analyses

In cross-sectional analyses, stratified by sex, we investigated associations between the flavan-3-ol biomarker, (specific gravity adjusted concentration, log2-transformed), as an independent variable and systolic and diastolic blood pressure (mmHg) using multiple regression analyses. Statistical models were selected a priori based on likely confounders. Model 0 was adjusted for age (continuous; years); model 1 additionally for BMI (continuous; kg/m$^2$) ; model 2: additionally for smoking status (categorical; never, ever, former), physical activity (categorical; inactive, moderately inactive, moderately active, active) and social class (categorical; unclassified, A, B, C1, C2, D, E); model 3: additionally for plasma vitamin C as marker of fruit and vegetable intake (continuous, $\upmu$M), tea and coffee intake (continuous, g/day, derived from 7-day diary); model 4: model 2, additionally adjusted for baseline health (self-reported diabetes mellitus, myocardial infarction, cerebrovascular accident), family history of myocardial infarction, use of anti-hypertensive or lipid-lowering drugs (all categorical; yes, no); model 5: model 3, additionally adjusted for baseline health (self-reported diabetes mellitus, myocardial infarction, cerebrovascular accident), family history of myocardial infarction, use of anti-hypertensive or lipid-lowering drugs (all categorical; yes, no). Analyses in women were additionally adjusted for menopausal status [categorical; pre-menopausal, peri-menopausal (2 categories), post-menopausal] and hormone replacement therapy (categorical; current, former, never).

### Association between biomarker and disease risk and mortality

In prospective analyses, we investigated associations between the flavan-3-ol biomarker, (specific gravity adjusted concentration, $\hbox {log}_2$-transformed), as an independent variable and disease risk (CVD, IHD, MI and Stroke), and mortality using Cox regression analyses. The proportional hazard assumptions has been tested using the *cox.zph* function and was met. Statistical models were selected a priori based on likely confounders. All models were stratified by age (as decade) at baseline, but age was not included as a covariable. Otherwise, models were the same as described for cross-sectional analyses.

### Sensitivity and complete cases analyses

Post-hoc sensitivity analyses were conducted by restricting the study population to those without any self-reported disease or disease risk at baseline and post-menopausal women. The same analyses as described above were also conducted on a subsample of the study population for whom all data were available (complete cases analysis).

## Supplementary information

Supplementary Information.
