# Supplementary material for: Biomarker-estimated flavan-3-ol intake is associated with lower blood pressure in cross-sectional analysis in EPIC Norfolk
Source: Sci Rep. 2020 Oct 21;10:17964. doi: 10.1038/s41598-020-74863-7 (PMC7578063; doi:10.1038/s41598-020-74863-7)
Supplement: Supplementary file 1 — Supplementary Information. [file 41598_2020_74863_MOESM1_ESM.pdf]

Biomarker estimated flavan-3-ol intake is associated with lower blood pressure in cross-sectional analysis in EPIC Norfolk

Supplemental material

Javier I. Ottaviani[1], Abigail Britten[2], Debora Lucarelli[2], Robert Luben[3], Angela A. Mulligan[2], Marleen A. Lentjes[4], Reedmond Fong[5], Nicola Gray[6], Philip B. Grace[7], Deborah H. Mawson[7], Amy Tym[7], Antonia Wierzbicki[7], Nita G. Forouhi[2], Kay-Tee Khaw[3], Hagen Schroeter[1], Gunter G. C. Kuhnle[6,*]

[1]Mars, Inc., McLean, VA; [2]MRC Epidemiology Unit, University of Cambridge, UK; [3]Department of Public Health and Primary Care, University of Cambridge, UK; [4]School of Medical Sciences, Örebro University, Sweden; [5]Department of Nutrition, UC Davis, CA; [6]Department of Food and Nutritional Sciences, University of Reading, UK;, [7] LGC, Newmarket Road, Fordham, UK;

*Corresponding author: Gunter G. C. Kuhnle; Department of Food & Nutritional Sciences, University of Reading, Reading RG6 6UR, UK. Telephone: +44 118 378 7723; Email: g.g.kuhnle@reading.ac.uk

Study population

Supplementary Table 1: Baseline characteristics, disease incidence and mortality of 25,618 participants of EPIC Norfolk. Data show mean (SD) or absolute number and percentage and number of participants with missing data.

	Overall	Missing	Complete	Men	Women
n	25618		18466	11592	14026
Age [years]	58.74 (9.32)	0	58.78 (9.03)	59.13 (9.31)	58.42 (9.31)
BMI [kg/m2]	26.36 (3.92)	57	26.24 (3.77)	26.53 (3.31)	26.23 (4.35)
Sex [Female]	14026 (54.8)	0	10017 (54.2)	—	14026 (100.0)
Physical activity		1			
Inactive	7853 (30.7)		5457 (29.6)	3579 (30.9)	4274 (30.5)
Moderately Inactive	7344 (28.7)		5322 (28.8)	2853 (24.6)	4491 (32.0)
Moderately Active	5773 (22.5)		4273 (23.1)	2657 (22.9)	3116 (22.2)
Active	4647 (18.1)		3414 (18.5)	2502 (21.6)	2145 (15.3)
Smoking status		220			
Current	2979 (11.7)		2091 (11.3)	1402 (12.2)	1577 (11.4)
Former	10751 (42.3)		7814 (42.3)	6276 (54.5)	4475 (32.2)
Never	11668 (45.9)		8561 (46.4)	3833 (33.3)	7835 (56.4)
Systolic BP [mmHg]	135.49 (18.46)	60	135.53 (18.24)	137.41 (17.69)	133.91 (18.93)
Diastolic BP [mmHg]	82.54 (11.28)	60	82.51 (11.18)	84.40 (11.13)	81.00 (11.16)
Total Cholesterol [mmol/L]	6.18 (1.17)	1765	6.19 (1.16)	6.04 (1.10)	6.30 (1.21)
LDL [mmol/L]	1.42 (0.43)	2562	1.42 (0.43)	1.23 (0.33)	1.57 (0.43)
HDL [mmol/L]	3.97 (1.04)	2561	3.97 (1.03)	3.92 (0.96)	4.01 (1.09)
Triglycerides [mmol/L]	1.82 (1.10)	1767	1.81 (1.10)	2.05 (1.22)	1.62 (0.96)
cRP	3.09 (6.27)	7188	3.00 (5.98)	3.02 (5.92)	3.15 (6.54)
Social class		488			
Unclassified	82 (0.3)		52 (0.3)	33 (0.3)	49 (0.4)
A	1750 (7.0)		1282 (6.9)	872 (7.6)	878 (6.4)
B	9152 (36.4)		6758 (36.6)	4353 (38.1)	4799 (35.0)
C1D	4138 (16.5)		3042 (16.5)	1424 (12.5)	2714 (19.8)
C2D	5767 (22.9)		4236 (22.9)	2881 (25.2)	2886 (21.0)
E	3356 (13.4)		2469 (13.4)	1519 (13.3)	1837 (13.4)
F	885 (3.5)		627 (3.4)	337 (3.0)	548 (4.0)
Plasma Vitamin C [µmol/L]	53.34 (20.37)	3161	53.29 (20.13)	47.07 (18.91)	58.61 (20.05)
Medication					
Lipid-lowering	377 (1.5)	0	265 (1.4)	173 (1.5)	204 (1.5)
Anti-hypertensive	4798 (18.7)	0	3422 (18.5)	2165 (18.7)	2633 (18.8)
Participants free from disease at baseline					
Diabetes	25018 (97.7)	19	18055 (97.8)	11222 (96.9)	13796 (98.4)
MI	24801 (96.9)	22	17881 (96.8)	10966 (94.7)	13835 (98.7)
CVA	25251 (98.6)	17	18224 (98.7)	11377 (98.2)	13874 (99.0)
Family history of MI	16280 (63.6)	24	11743 (63.6)	7466 (64.5)	8814 (62.9)
Tea intake (7DD) [g/d]	772.48 (530.58)	132	777.12 (525.95)	802.69 (556.57)	747.56 (506.81)
Coffee intake (7DD) [g/d]	385.53 (410.93)	132	394.78 (411.68)	394.20 (423.18)	378.38 (400.41)
Menopausal status		15			

Pre-menopausal	2349 (16.8)		1594 (15.9)	—	2349 (16.8)
Peri-menopausal (< 1year)	760 (5.4)		553 (5.5)	—	760 (5.4)
Peri-menopausal (1-5 years)	2510 (17.9)		1864 (18.6)	—	2510 (17.9)
Post-menopausal	8392 (59.9)		6003 (59.9)	—	8392 (59.9)
Hormone replacement therapy		15			
Current	2847 (20.3)		2100 (21.0)	—	2847 (20.3)
Former	1590 (11.3)		1127 (11.3)	—	1590 (11.3)
Never	9574 (68.3)		6787 (67.8)	—	9574 (68.3)
Biomarker					
gVLM [µmol/L]	9.51 (15.98)	1466	9.63 (16.21)	10.48 (16.51)	8.71 (15.49)
gVLM (adjusted by specific gravity)	9.32 (15.69)	3779	9.45 (15.87)	10.20 (16.17)	8.59 (15.25)
SREM [µmol/L]	1.97 (3.01)	1326	1.93 (2.95)	2.15 (3.01)	1.82 (3.00)
SREM (adjusted by specific gravity)	1.90 (2.92)	3655	1.89 (2.88)	2.08 (2.91)	1.76 (2.92)
Specific gravity	1.02 (0.01)	2838	1.02 (0.01)	1.02 (0.01)	1.02 (0.01)
Disease incidence at end of follow-up		0			
CVD	13969 (54.5)		10044 (54.4)	6907 (59.6)	7062 (50.3)
Mortality at end of follow-up		0			
All cause	8030 (31.3)		5633 (30.5)	4277 (36.9)	3753 (26.8)
CVD	2613 (10.2)		1807 (9.8)	1474 (12.7)	1139 (8.1)
IHD	1278 (5.0)		869 (4.7)	858 (7.4)	420 (3.0)
MI	492 (1.9)		328 (1.8)	319 (2.8)	173 (1.2)

Supplementary Table 2: Baseline characteristics of 25,618 participants of EPIC Norfolk and number of missing data, stratified by biomarker concentration. Data show mean (SD) or absolute number and percentage. Differences between centiles were tested by ANOVA (continuous data) or chi2-test.

Centiles	0-10	10-25	25-50	50-75	75-90	90-100	p
Biomarker range (log_2-transformed)	-9.795,-2.505	-2.505,-0.288	-0.288, 1.673	1.673, 3.351	3.351, 4.677	4.677, 6.907	
n	2183	3276	5460	5459	3277	2183	
Sex [Female]	1367 (62.6)	1966 (60.0)	3063 (56.1)	2892 (53.0)	1617 (49.3)	1084 (49.7)	<0.001
Age [years]	58.49 (8.90)	59.17 (8.91)	59.24 (9.13)	59.24 (9.05)	58.69 (9.26)	58.08 (9.25)	<0.001
BMI [kg/m2]	26.03 (3.80)	26.06 (3.85)	26.34 (3.84)	26.37 (3.88)	26.56 (3.91)	26.61 (3.98)	<0.001
Physical activity							0.108
Inactive	635 (29.1)	1005 (30.7)	1708 (31.3)	1646 (30.2)	1015 (31.0)	655 (30.0)	
Moderately Inactive	655 (30.0)	972 (29.7)	1598 (29.3)	1539 (28.2)	899 (27.4)	605 (27.7)	
Moderately Active	510 (23.4)	746 (22.8)	1211 (22.2)	1258 (23.0)	742 (22.6)	488 (22.4)	
Active	383 (17.5)	553 (16.9)	943 (17.3)	1016 (18.6)	621 (19.0)	435 (19.9)	
Smoking status							<0.001
Current	288 (13.3)	347 (10.7)	586 (10.8)	616 (11.4)	416 (12.8)	278 (12.8)	
Former	854 (39.4)	1311 (40.4)	2245 (41.4)	2309 (42.7)	1439 (44.3)	983 (45.3)	
Never	1027 (47.3)	1587 (48.9)	2586 (47.7)	2483 (45.9)	1391 (42.9)	911 (41.9)	
Systolic BP [mmHg]	136.77 (19.09)	136.29 (18.94)	135.90 (18.58)	136.38 (18.46)	135.26 (18.01)	133.62 (17.30)	<0.001
Diastolic BP [mmHg]	83.30 (11.44)	82.80 (11.51)	82.70 (11.22)	82.92 (11.40)	82.26 (10.98)	81.77 (11.05)	<0.001
Total Cholesterol [mmol/L]	6.24 (1.21)	6.24 (1.15)	6.23 (1.18)	6.19 (1.16)	6.14 (1.14)	6.12 (1.16)	<0.001
LDL [mmol/L]	1.48 (0.43)	1.45 (0.42)	1.42 (0.41)	1.42 (0.45)	1.38 (0.40)	1.40 (0.42)	<0.001
HDL [mmol/L]	4.01 (1.07)	4.03 (1.03)	4.01 (1.04)	3.95 (1.03)	3.93 (1.03)	3.89 (1.00)	<0.001
Triglycerides [mmol/L]	1.70 (1.19)	1.71 (0.98)	1.81 (1.07)	1.86 (1.16)	1.89 (1.07)	1.87 (1.12)	<0.001
cRP	3.12 (6.67)	2.92 (6.27)	3.03 (6.06)	3.04 (5.58)	3.18 (7.00)	2.89 (4.36)	0.596
Social class							<0.001
Unclassified	6 (0.3)	14 (0.4)	11 (0.2)	12 (0.2)	16 (0.5)	4 (0.2)	
A	191 (8.9)	231 (7.2)	398 (7.4)	342 (6.4)	202 (6.3)	125 (5.8)	

B	800 (37.2)	1204 (37.6)	1929 (36.0)	1904 (35.6)	1170 (36.4)	773 (36.0)	
C1D	379 (17.6)	549 (17.1)	890 (16.6)	866 (16.2)	510 (15.9)	339 (15.8)	
C2D	457 (21.2)	716 (22.4)	1227 (22.9)	1246 (23.3)	757 (23.5)	526 (24.5)	
E	269 (12.5)	386 (12.1)	711 (13.3)	773 (14.4)	439 (13.6)	305 (14.2)	
F	51 (2.4)	102 (3.2)	188 (3.5)	209 (3.9)	123 (3.8)	78 (3.6)	
Plasma Vitamin C [µmol/L]	55.48 (21.43)	54.09 (20.33)	53.52 (20.34)	52.82 (19.91)	51.76 (19.95)	52.02 (19.38)	<0.001
Tea intake (7DD) [g/d]	640.34 (524.62)	734.64 (544.88)	779.40 (524.49)	807.05 (525.18)	820.27 (511.60)	813.27 (514.15)	<0.001
Coffee intake (7DD) [g/d]	521.48 (516.12)	426.34 (424.40)	390.61 (403.83)	365.46 (385.62)	350.70 (369.21)	336.45 (377.92)	<0.001
gVLM [µmol/L]	0.06 (0.06)	0.47 (0.19)	1.83 (0.69)	6.11 (2.03)	16.48 (4.43)	49.68 (21.30)	<0.001
gVLM (adjusted by specific gravity)	0.06 (0.06)	0.47 (0.19)	1.80 (0.68)	6.01 (1.99)	16.17 (4.34)	48.66 (20.83)	<0.001
SREM [µmol/L]	0.62 (1.24)	1.01 (1.84)	1.45 (2.25)	1.97 (2.76)	2.83 (3.51)	4.21 (4.41)	<0.001
SREM (adjusted by specific gravity)	0.61 (1.22)	1.00 (1.80)	1.42 (2.21)	1.93 (2.70)	2.77 (3.43)	4.12 (4.31)	<0.001

SREM concentration

Biomarker range (log-transformed)	-9.795,-2.505	-2.505,-0.288	-0.288, 1.673	1.673, 3.351	3.351, 4.677	4.677, 6.907	p
n	2183	3276	5460	5459	3277	2183	
Sex [Female]	1367 (62.6)	1966 (60.0)	3063 (56.1)	2892 (53.0)	1617 (49.3)	1084 (49.7)	<0.001
Age [years]	58.49 (8.90)	59.17 (8.91)	59.24 (9.13)	59.24 (9.05)	58.69 (9.26)	58.08 (9.25)	<0.001
BMI [kg/m2]	26.03 (3.80)	26.06 (3.85)	26.34 (3.84)	26.37 (3.88)	26.56 (3.91)	26.61 (3.98)	<0.001
Physical activity							0.108
Inactive	635 (29.1)	1005 (30.7)	1708 (31.3)	1646 (30.2)	1015 (31.0)	655 (30.0)	
Moderately Inactive	655 (30.0)	972 (29.7)	1598 (29.3)	1539 (28.2)	899 (27.4)	605 (27.7)	
Moderately Active	510 (23.4)	746 (22.8)	1211 (22.2)	1258 (23.0)	742 (22.6)	488 (22.4)	
Active	383 (17.5)	553 (16.9)	943 (17.3)	1016 (18.6)	621 (19.0)	435 (19.9)	
Smoking status							<0.001
Current	288 (13.3)	347 (10.7)	586 (10.8)	616 (11.4)	416 (12.8)	278 (12.8)	
Former	854 (39.4)	1311 (40.4)	2245 (41.4)	2309 (42.7)	1439 (44.3)	983 (45.3)	

Never	1027 (47.3)	1587 (48.9)	2586 (47.7)	2483 (45.9)	1391 (42.9)	911 (41.9)	
Systolic BP [mmHg]	136.77 (19.09)	136.29 (18.94)	135.90 (18.58)	136.38 (18.46)	135.26 (18.01)	133.62 (17.30)	<0.001
Diastolic BP [mmHg]	83.30 (11.44)	82.80 (11.51)	82.70 (11.22)	82.92 (11.40)	82.26 (10.98)	81.77 (11.05)	<0.001
Total Cholesterol [mmol/L]	6.24 (1.21)	6.24 (1.15)	6.23 (1.18)	6.19 (1.16)	6.14 (1.14)	6.12 (1.16)	<0.001
LDL [mmol/L]	1.48 (0.43)	1.45 (0.42)	1.42 (0.41)	1.42 (0.45)	1.38 (0.40)	1.40 (0.42)	<0.001
HDL [mmol/L]	4.01 (1.07)	4.03 (1.03)	4.01 (1.04)	3.95 (1.03)	3.93 (1.03)	3.89 (1.00)	<0.001
Triglycerides [mmol/L]	1.70 (1.19)	1.71 (0.98)	1.81 (1.07)	1.86 (1.16)	1.89 (1.07)	1.87 (1.12)	<0.001
cRP	3.12 (6.67)	2.92 (6.27)	3.03 (6.06)	3.04 (5.58)	3.18 (7.00)	2.89 (4.36)	0.596
Social class							<0.001
Unclassified	6 (0.3)	14 (0.4)	11 (0.2)	12 (0.2)	16 (0.5)	4 (0.2)	
A	191 (8.9)	231 (7.2)	398 (7.4)	342 (6.4)	202 (6.3)	125 (5.8)	
B	800 (37.2)	1204 (37.6)	1929 (36.0)	1904 (35.6)	1170 (36.4)	773 (36.0)	
C1D	379 (17.6)	549 (17.1)	890 (16.6)	866 (16.2)	510 (15.9)	339 (15.8)	
C2D	457 (21.2)	716 (22.4)	1227 (22.9)	1246 (23.3)	757 (23.5)	526 (24.5)	
E	269 (12.5)	386 (12.1)	711 (13.3)	773 (14.4)	439 (13.6)	305 (14.2)	
F	51 (2.4)	102 (3.2)	188 (3.5)	209 (3.9)	123 (3.8)	78 (3.6)	
Plasma Vitamin C [µmol/L]	55.48 (21.43)	54.09 (20.33)	53.52 (20.34)	52.82 (19.91)	51.76 (19.95)	52.02 (19.38)	<0.001
Tea intake (7DD) [g/d]	640.34 (524.62)	734.64 (544.88)	779.40 (524.49)	807.05 (525.18)	820.27 (511.60)	813.27 (514.15)	<0.001
Coffee intake (7DD) [g/d]	521.48 (516.12)	426.34 (424.40)	390.61 (403.83)	365.46 (385.62)	350.70 (369.21)	336.45 (377.92)	<0.001
gVLM [µmol/L]	0.06 (0.06)	0.47 (0.19)	1.83 (0.69)	6.11 (2.03)	16.48 (4.43)	49.68 (21.30)	<0.001
gVLM (adjusted by specific gravity)	0.06 (0.06)	0.47 (0.19)	1.80 (0.68)	6.01 (1.99)	16.17 (4.34)	48.66 (20.83)	<0.001
SREM [µmol/L]	0.62 (1.24)	1.01 (1.84)	1.45 (2.25)	1.97 (2.76)	2.83 (3.51)	4.21 (4.41)	<0.001
SREM (adjusted by specific gravity)	0.61 (1.22)	1.00 (1.80)	1.42 (2.21)	1.93 (2.70)	2.77 (3.43)	4.12 (4.31)	<0.001

Supplemental Table 3: **Associations between biomarker-estimated flavan-3-ol intake and CVD risk markers, comparison of full dataset and complete cases (CC) analysis.** Data shown are estimated differences (95% CI) between low (10th centile) and high (90th centile) of estimated, using different statistical models†.

	Men				Women‡			
	Systol		Diastol		Systol		Diastol	
	Full dataset	CC	Full dataset	CC	Full dataset	CC	Full dataset	CC
gVLM¶								
Model 0	-1.6 (-2.4; -0.8)	-1.6 (-2.5; -0.7)	-1.0 (-1.5; -0.4)	-1.0 (-1.6; -0.5)	-1.8 (-2.5; -1.0)	-1.8 (-2.6; -1.0)	-1.1 (-1.6; -0.6)	-1.1 (-1.7; -0.6)
Model 1	-1.8 (-2.6; -1.0)	-1.8 (-2.7; -0.9)	-1.2 (-1.7; -0.7)	-1.2 (-1.8; -0.6)	-2.2 (-3.0; -1.5)	-2.2 (-3.1; -1.4)	-1.4 (-1.9; -0.9)	-1.5 (-2.0; -0.9)
Model 2	-1.8 (-2.7; -1.0)	-1.8 (-2.7; -0.9)	-1.2 (-1.7; -0.6)	-1.2 (-1.8; -0.6)	-2.3 (-3.0; -1.5)	-2.3 (-3.1; -1.5)	-1.4 (-1.9; -0.9)	-1.5 (-2.0; -1.0)
Model 3	-2.0 (-2.8; -1.2)	-1.9 (-2.8; -1.0)	-1.3 (-1.8; -0.7)	-1.3 (-1.9; -0.7)	-2.6 (-3.3; -1.8)	-2.5 (-3.4; -1.7)	-1.6 (-2.0; -1.1)	-1.6 (-2.1; -1.1)
Model 4	-1.8 (-2.6; -1.0)	-1.8 (-2.7; -0.9)	-1.2 (-1.7; -0.7)	-1.2 (-1.8; -0.6)	-2.3 (-3.0; -1.5)	-2.2 (-3.1; -1.4)	-1.4 (-1.9; -0.9)	-1.5 (-2.0; -1.0)
Model 5	-1.9 (-2.7; -1.1)	-1.9 (-2.8; -1.0)	-1.3 (-1.8; -0.7)	-1.3 (-1.9; -0.7)	-2.5 (-3.3; -1.8)	-2.5 (-3.3; -1.6)	-1.6 (-2.0; -1.1)	-1.6 (-2.1; -1.1)
SREM¶								
Model 0	-1.8 (-2.6; -0.9)	-1.5 (-2.4; -0.6)	-0.7 (-1.3; -0.2)	-0.6 (-1.2; 0.0)	-1.3 (-2.1; -0.5)	-1.4 (-2.2; -0.5)	-0.3 (-0.8; 0.2)	-0.3 (-0.9; 0.2)
Model 1	-2.0 (-2.8; -1.2)	-1.7 (-2.6; -0.8)	-0.9 (-1.4; -0.4)	-0.8 (-1.4; -0.2)	-1.9 (-2.7; -1.1)	-1.9 (-2.8; -1.1)	-0.8 (-1.2; -0.3)	-0.7 (-1.3; -0.2)
Model 2	-2.1 (-2.9; -1.2)	-1.8 (-2.7; -0.9)	-0.9 (-1.4; -0.4)	-0.8 (-1.4; -0.2)	-2.0 (-2.7; -1.2)	-2.0 (-2.9; -1.2)	-0.8 (-1.3; -0.3)	-0.8 (-1.3; -0.2)
Model 3	-2.5 (-3.3; -1.6)	-2.3 (-3.3; -1.4)	-1.2 (-1.7; -0.6)	-1.1 (-1.8; -0.5)	-2.9 (-3.7; -2.1)	-2.8 (-3.7; -1.9)	-1.2 (-1.7; -0.7)	-1.1 (-1.7; -0.5)
Model 4	-2.0 (-2.8; -1.2)	-1.8 (-2.7; -0.9)	-0.9 (-1.4; -0.4)	-0.8 (-1.4; -0.2)	-1.9 (-2.7; -1.1)	-2.0 (-2.8; -1.1)	-0.8 (-1.3; -0.3)	-0.8 (-1.3; -0.2)
Model 5	-2.4 (-3.3; -1.5)	-2.3 (-3.2; -1.3)	-1.2 (-1.7; -0.6)	-1.1 (-1.8; -0.5)	-2.8 (-3.6; -2.0)	-2.7 (-3.6; -1.8)	-1.2 (-1.7; -0.6)	-1.1 (-1.7; -0.5)

†) Model 0: adjusted for age; model 1: additionally adjusted for BMI; model 2: additionally adjusted for smoking status, physical activity and social class; model 3: additionally adjusted plasma vitamin C as marker of fruit and vegetable intake, tea and coffee intake; model 4: model 2, additionally adjusted for baseline health (self-reported diabetes mellitus, myocardial infarction, cerebrovascular accident), family history of myocardial infarction, use of anti-hypertensive or lipid-lowering drugs; model 5: model 3, additionally adjusted for baseline health (self-reported diabetes mellitus, myocardial infarction, cerebrovascular accident), family history of myocardial infarction, use of anti-hypertensive or lipid-lowering drugs. ‡) additionally adjusted for menopausal status and hormone replacement therapy; ¶) biomarker concentrations were adjusted by specific gravity

Supplemental Table 4: **Associations between biomarker-estimated flavan-3-ol intake and CVD risk markers, comparison of full dataset and complete cases (CC) analysis**. Data shown are estimated differences (95% CI) between low (10th centile) and high (90th centile) of estimated intake, using different statistical models†

	Cholesterol		HDL		LDL		Triglycerides		CRP	
	Full dataset	CC	Full dataset	CC	Full dataset	CC	Full dataset	CC	Full dataset	CC
Men										
gVLM¶										
Model 0	-0.1 (-0.1; 0.0)	-0.1 (-0.1; 0.0)	0.0 (0.0; 0.0)	0.0 (0.0; 0.0)	-0.1 (-0.1; 0.0)	-0.1 (-0.1; 0.0)	0.1 (0.0; 0.1)	0.1 (0.0; 0.1)	-0.1 (-0.5; 0.2)	-0.1 (-0.4; 0.2)
Model 1	-0.1 (-0.1; 0.0)	-0.1 (-0.1; 0.0)	0.0 (0.0; 0.0)	0.0 (0.0; 0.0)	-0.1 (-0.1; 0.0)	-0.1 (-0.1; 0.0)	0.1 (0.0; 0.1)	0.1 (0.0; 0.1)	-0.2 (-0.5; 0.2)	-0.1 (-0.4; 0.2)
Model 2	-0.1 (-0.1; 0.0)	-0.1 (-0.1; 0.0)	0.0 (0.0; 0.0)	0.0 (0.0; 0.0)	-0.1 (-0.1; 0.0)	-0.1 (-0.1; 0.0)	0.1 (0.0; 0.1)	0.1 (0.0; 0.1)	-0.2 (-0.5; 0.1)	-0.2 (-0.5; 0.1)
Model 3	-0.1 (-0.1; 0.0)	-0.1 (-0.1; 0.0)	0.0 (0.0; 0.0)	0.0 (0.0; 0.0)	-0.1 (-0.1; 0.0)	-0.1 (-0.1; 0.0)	0.0 (0.0; 0.1)	0.0 (0.0; 0.1)	-0.2 (-0.6; 0.1)	-0.2 (-0.5; 0.1)
Model 4	-0.1 (-0.1; 0.0)	-0.1 (-0.1; 0.0)	0.0 (0.0; 0.0)	0.0 (0.0; 0.0)	-0.1 (-0.1; 0.0)	-0.1 (-0.1; 0.0)	0.1 (0.0; 0.1)	0.1 (0.0; 0.1)	-0.2 (-0.5; 0.2)	-0.2 (-0.5; 0.1)
Model 5	-0.1 (-0.1; 0.0)	-0.1 (-0.1; 0.0)	0.0 (0.0; 0.0)	0.0 (0.0; 0.0)	-0.1 (-0.1; 0.0)	-0.1 (-0.1; 0.0)	0.1 (0.0; 0.1)	0.1 (0.0; 0.1)	-0.2 (-0.5; 0.1)	-0.2 (-0.5; 0.1)
SREM¶										
Model 0	-0.1 (-0.2; -0.1)	-0.1 (-0.2; -0.1)	0.0 (0.0; 0.0)	0.0 (0.0; 0.0)	-0.2 (-0.2; -0.1)	-0.2 (-0.2; -0.1)	0.1 (0.1; 0.2)	0.1 (0.1; 0.2)	0.2 (-0.2; 0.5)	0.3 (0.0; 0.6)
Model 1	-0.1 (-0.2; -0.1)	-0.1 (-0.2; -0.1)	0.0 (0.0; 0.0)	0.0 (0.0; 0.0)	-0.2 (-0.2; -0.1)	-0.2 (-0.2; -0.1)	0.1 (0.1; 0.2)	0.1 (0.1; 0.2)	0.1 (-0.2; 0.5)	0.2 (-0.1; 0.5)
Model 2	-0.1 (-0.2; -0.1)	-0.1 (-0.2; -0.1)	0.0 (0.0; 0.0)	0.0 (0.0; 0.0)	-0.2 (-0.2; -0.1)	-0.2 (-0.2; -0.1)	0.1 (0.1; 0.2)	0.1 (0.1; 0.2)	0.1 (-0.3; 0.4)	0.2 (-0.1; 0.5)
Model 3	-0.1 (-0.1; 0.0)	-0.1 (-0.2; 0.0)	0.0 (0.0; 0.0)	0.0 (0.0; 0.0)	-0.1 (-0.2; -0.1)	-0.2 (-0.2; -0.1)	0.1 (0.0; 0.2)	0.1 (0.0; 0.2)	0.0 (-0.4; 0.3)	0.1 (-0.2; 0.4)
Model 4	-0.1 (-0.2; -0.1)	-0.1 (-0.2; -0.1)	0.0 (0.0; 0.0)	0.0 (0.0; 0.0)	-0.2 (-0.2; -0.1)	-0.2 (-0.2; -0.1)	0.1 (0.1; 0.2)	0.1 (0.1; 0.2)	0.1 (-0.3; 0.4)	0.2 (-0.1; 0.5)
Model 5	-0.1 (-0.1; 0.0)	-0.1 (-0.2; 0.0)	0.0 (0.0; 0.0)	0.0 (0.0; 0.0)	-0.1 (-0.2; -0.1)	-0.2 (-0.2; -0.1)	0.1 (0.1; 0.2)	0.1 (0.1; 0.2)	0.0 (-0.3; 0.4)	0.1 (-0.2; 0.5)
Women‡										
gVLM¶										
Model 0	-0.1 (-0.1; 0.0)	-0.1 (-0.1; 0.0)	0.0 (-0.1; 0.0)	0.0 (-0.1; 0.0)	-0.1 (-0.1; 0.0)	-0.1 (-0.1; 0.0)	0.1 (0.1; 0.2)	0.1 (0.1; 0.2)	0.3 (0.0; 0.6)	0.3 (0.0; 0.7)
Model 1	-0.1 (-0.1; 0.0)	-0.1 (-0.1; 0.0)	0.0 (0.0; 0.0)	0.0 (0.0; 0.0)	-0.1 (-0.1; 0.0)	-0.1 (-0.1; 0.0)	0.1 (0.0; 0.1)	0.1 (0.0; 0.1)	0.2 (-0.1; 0.5)	0.2 (-0.2; 0.5)
Model 2	-0.1 (-0.1; 0.0)	-0.1 (-0.1; 0.0)	0.0 (0.0; 0.0)	0.0 (0.0; 0.0)	-0.1 (-0.1; 0.0)	-0.1 (-0.1; 0.0)	0.1 (0.0; 0.1)	0.1 (0.0; 0.1)	0.2 (-0.1; 0.5)	0.2 (-0.2; 0.5)
Model 3	-0.1 (-0.1; 0.0)	-0.1 (-0.1; 0.0)	0.0 (0.0; 0.0)	0.0 (0.0; 0.0)	-0.1 (-0.1; 0.0)	-0.1 (-0.1; 0.0)	0.1 (0.0; 0.1)	0.1 (0.0; 0.1)	0.1 (-0.2; 0.4)	0.1 (-0.2; 0.5)
Model 4	-0.1 (-0.1; 0.0)	-0.1 (-0.1; 0.0)	0.0 (0.0; 0.0)	0.0 (0.0; 0.0)	-0.1 (-0.1; 0.0)	-0.1 (-0.1; 0.0)	0.1 (0.0; 0.1)	0.1 (0.0; 0.1)	0.2 (-0.1; 0.5)	0.2 (-0.1; 0.6)
Model 5	-0.1 (-0.1; 0.0)	-0.1 (-0.1; 0.0)	0.0 (0.0; 0.0)	0.0 (0.0; 0.0)	-0.1 (-0.1; 0.0)	-0.1 (-0.1; 0.0)	0.1 (0.0; 0.1)	0.1 (0.0; 0.1)	0.1 (-0.2; 0.4)	0.1 (-0.2; 0.5)
SREM¶										
Model 0	0.0 (-0.1; 0.1)	0.0 (0.0; 0.1)	0.0 (0.0; 0.0)	0.0 (0.0; 0.0)	0.0 (-0.1; 0.0)	0.0 (-0.1; 0.0)	0.2 (0.1; 0.2)	0.2 (0.1; 0.2)	0.4 (0.1; 0.7)	0.5 (0.1; 0.8)
Model 1	0.0 (-0.1; 0.0)	0.0 (-0.1; 0.0)	0.0 (0.0; 0.0)	0.0 (0.0; 0.0)	-0.1 (-0.1; 0.0)	-0.1 (-0.1; 0.0)	0.1 (0.1; 0.2)	0.1 (0.1; 0.2)	0.3 (0.0; 0.6)	0.3 (0.0; 0.7)
Model 2	0.0 (-0.1; 0.0)	0.0 (-0.1; 0.0)	0.0 (0.0; 0.0)	0.0 (0.0; 0.0)	-0.1 (-0.1; 0.0)	-0.1 (-0.1; 0.0)	0.1 (0.1; 0.2)	0.1 (0.1; 0.2)	0.3 (0.0; 0.6)	0.3 (0.0; 0.7)
Model 3	0.0 (-0.1; 0.1)	0.0 (-0.1; 0.1)	0.0 (0.0; 0.0)	0.0 (0.0; 0.0)	-0.1 (-0.1; 0.0)	-0.1 (-0.1; 0.0)	0.1 (0.1; 0.1)	0.1 (0.1; 0.2)	0.1 (-0.3; 0.4)	0.1 (-0.3; 0.5)
Model 4	0.0 (-0.1; 0.0)	0.0 (-0.1; 0.0)	0.0 (0.0; 0.0)	0.0 (0.0; 0.0)	-0.1 (-0.1; 0.0)	-0.1 (-0.1; 0.0)	0.1 (0.1; 0.2)	0.1 (0.1; 0.2)	0.3 (0.0; 0.6)	0.3 (0.0; 0.7)
Model 5	0.0 (-0.1; 0.1)	0.0 (-0.1; 0.1)	0.0 (0.0; 0.0)	0.0 (0.0; 0.0)	-0.1 (-0.1; 0.0)	-0.1 (-0.1; 0.0)	0.1 (0.1; 0.1)	0.1 (0.1; 0.2)	0.1 (-0.2; 0.4)	0.1 (-0.3; 0.5)

†) Model 0: adjusted for age; model 1: additionally adjusted for BMI; model 2: additionally adjusted for smoking status, physical activity and social class; model 3: additionally adjusted plasma vitamin C as marker of fruit and vegetable intake, tea and coffee intake; model 4: model 2, additionally adjusted for baseline health (self-reported diabetes mellitus, myocardial infarction, cerebrovascular accident), family history of myocardial infarction, use of anti-hypertensive or lipid-lowering drugs; model 5: model 3, additionally adjusted for baseline health (self-reported diabetes mellitus, myocardial infarction, cerebrovascular accident), family history of myocardial infarction, use of anti-hypertensive or lipid-lowering drugs. ‡) additionally adjusted for menopausal status and hormone replacement therapy; ¶) biomarker concentrations were adjusted by specific gravity
